# Genetic analysis of reproductive performance of Frieswal cattle at Military Farm, Ambala

**DOI:** 10.14202/vetworld.2015.1032-1037

**Published:** 2015-09-08

**Authors:** Jagdeep Kumar, Y. P. Singh, Sushil Kumar, Rajbir Singh, Ravinder Kumar, Pradeep Kumar

**Affiliations:** 1 Department of Animal Husbandry, College of Veterinary and Animal Sciences, Sardar Vallabhbhai Patel University of Agriculture & Technology, Meerut, Uttar Pradesh, India; 2 Dairy Cattle Breeding Division, Central Institute for Research on Cattle, Meerut, Uttar Pradesh, India

**Keywords:** Frieswal cattle, non-genetics factors, age at first calving, service period

## Abstract

**Aim:**

This study was carried out to investigate the genetic analysis of reproductive performance of Frieswal cattle at Military Farm, Ambala.

**Materials and Methods:**

A total number of 3005 lactation records of 1147 Frieswal cows over a period of 15 years extending from 1993 to 2007 were used to study at Military Dairy Farm, Ambala. The study period was divided into 5 period of 3 years each. The average performances of reproduction traits, effect of genetic and non-genetic factors were analyzed, and estimation of genetic and phenotypic parameters of reproduction traits was undertaken.

**Results:**

The age at first calving (AFC) differed significantly across the periods of calving. The AFC was lowest during the third period (1999-2001) and longest in the first period (1993-95). The effect of season and period of calving, lactation order and regression of AFC on dry period, calving interval and service period was highly significant. The effect of sire was non-significant. The heritability estimates were low for almost all the traits under study. The service period had a high genetic correlation with dry period and calving interval. The dry period also found to have a low genetic correlation with calving interval in Frieswal cows. Service period had a high phenotypic correlation with dry period and very high with a calving interval. The phenotypic correlation between the dry period and calving interval was recognized high.

**Conclusions:**

Low heritability estimate for the reproduction traits indicates that there is a very little additive genetic variance in these traits, and individual selection will not be helpful for improving them. Improvement may be brought through better feeding and management of cows by reducing the environmental variability.

## Introduction

In the last four decades, Indian dairy sector has undergone a major shift, mainly due to the synthesis of high yielding crossbred strains, which are playing a very good role for increasing milk production of the country. Consequently, milk production in the country has increased over 5-fold (from mere 121.8 million tons in 2010-2011 to 127.9 million tons in 2011-2012) [1]. Beside of this impressive growth in milk production and highest milk producer of the world, there is a shortage of milk due to increasing more affluent population of urban. At present, per capita availability of milk is about 290 g/day, which is higher than the recommendations of Indian Council of Medical Research [1].

The estimated demand for milk by the year 2022 in India would be about 210 million tons [2]. The total milk production (i.e. 127.9 million tons) of India is contributed by 77 million lactating animals (35.6 million buffaloes, 30.7 million indigenous and 10.7 million crossbred cattle [1]. Of the total milk produced in the country, 53.4, 22.9, 20.2 and 3.5% is shared by buffaloes, crossbred cattle, indigenous cattle and goat respectively [3]. This increase in milk production in India has been mainly due to increase in the population of breedable cross bred cows (6.03 million vs. 14.06) and buffaloes (42.35 vs. 57.87 million) during the period 1987 to 2007.

The success of a dairy herd depends on the level of reproduction performance of the animals. Milk production of a cow is a function of its genotype and the environment under which the animal is brought and maintained at given time and age. There are many non-genetic factors, which introduce bias in the estimation of genetic parameters. In the absence of accurate value of these traits, it becomes difficult to estimate the genetic parameters of the traits, which determine the optimum selection criteria for planned improvement of the crossbred animals [4]. In view of the above facts, this study was conducted on the Frieswal cows maintained at Military Farm, Ambala.

## Materials and Methods

### Study area

Ambala district of Haryana lies between 30° 10′:31° 35′ north latitudes and 76° 30′:77° 10′ east longitudes. The climate of Ambala district can be classified as subtropical monsoon, mild and dry winter, hot summer and sub-humid which is mainly dry with very hot summer and cold winter except during monsoon season when moist air of oceanic origin penetrate into the district. The hot weather season starts from mid-March to last week of the June followed by the south-west monsoon, which lasts up to September. The transition period from September to November forms the post monsoon season. The winter season starts late in November and remains up to first week of March. The normal annual rainfall of the district is 1076 mm and is unevenly distributed over the area. The average rainy days are 44. The south west monsoon sets in from last week of June and withdraws in the end of September, contributing about 81% of normal annual rainfall. July and August are the wettest months. Rest, 19% rainfall, is received during the non-monsoon period in the wake of western disturbances and thunderstorms. Generally, rainfall in the district increases from southwest to northeast. The mean maximum temperature is 40.8°C (May and June) and mean minimum is 6.8°C (January) of the district [5].

### Data collection and analysis

The data on reproduction traits, age at first calving (AFC), service period, dry period, and calving interval. Pertained to 1147 Frieswal cattle progeny of 64 Sires, over a period of 15 years (1993-2007). The total years were classified into five periods taking into three seasons Winter (November-February), Summer (March-June), Rainy (July-October) in accordance with agro-climatic condition of the study center. Generally, rainfall in the district increases from southwest to northeast. As the data were non-orthogonal with disproportionate sub-class numbers. The least squares means, heritability, genetic and phenotypic correlation and effects of genetic and non-genetic factors were estimated by using mixed model least squares and maximum likelihood program as suggested, by Harvey [6].

1.0. The following least squares model was used separately for analysis of variance of AFC:

Y_ijkl_ = µ + S_i_ + SB_j_+PB_k_ +e_ijkl_

Where,

Y_ijkl_ = observation on l^th^ animal of i^th^ sire of j^th^ season of birth and k^th^ period of birth.

µ = overall population mean

S_i_ = random effect due to i^th^ sire

SB_j_ = fixed effect of j^th^ season of birth

PB_k_ = fixed effect due to k^th^ period of birth

e_ijkl_ = random error assumed to be normally and independently distributed with mean zero and variance σ^2^_e_.

The least squares model was used for remaining traits, i.e. dry period, calving interval and service period separately as given below:

Where,

Y_ijklm_ = is the observation on m^th^ animal of i^th^ sire in j^th^ season of calving of k^th^ period of calving and l^th^ lactation order.

µ = population mean

S_i_ = random effect due to i^th^ sire

SC_j_ = fixed effect of j^th^ season of calving

PC_k_ = fixed effect of k^th^ period of calving

LO_l_ = fixed effect of l^th^ lactation order

b_1_ = Linear regression of AFC on production and reproduction traits

b_2_ =Quadratic regression (Q) of age of first calving on production and reproduction trait

e_ijklm_ = random error

## Results and Discussion

The overall least squares mean (Table-1) for AFC was estimated to be 980.41±8.22 days and found to be in accordance with the reports of Yadav *et al*. [7] however, Hassan and Khan [8] reported higher AFC. The effect of season of calving on AFC was non significant. Animals born in rainy season had lowest (968.65±10.72 days) average AFC while those born in winter season had highest (987.92±10.25 days) average AFC (Table-1). Non-significant effect of season of birth on AFC was observed in previous studies [7,9,10]. The average AFC was found to be significantly different in difference periods. The animals those born during the third period (1999-2001) showed lower (925.54±12.16 days) average AFC (Table-1). This variation indicates that AFC may be optimized with improvement in management. Singh; Dubey and Singh; Singh *et al*. [9,10,11] also observed a significant effect of a period of birth on average AFC. However, Singh *et al*. [11] could not find the significant effect of period of calving on AFC. Statistical analysis of variance (Table-2) showed that the AFC had non-significant effect on lactation length. Similar finding was also reported in the Annual Report of Project Directorate on Cattle [12].

**Table-1:** Least square means of AFC.

Factor	AFC	
	
Parameter	Number of observations	Least squares mean±SE (days)
Overall mean (µ)	1147	980.41±8.22
Period of calving		
Period 1 (1993-1995)	162	1064.38±19.51
Period 2 (1996-1998)	211	983.83±15.21
Period 3 (1999-2001)	429	925.54±12.16
Period 4 (2002-2004)	219	976.49±14.24
Period 5 (2005-2007)	126	951.82±18.24
Season of calving		
Winter (November-February)	478	987.92±10.25
Summer (March-June)	227	984.67±13.05
Rainy (July-October)	442	968.65±10.72

AFC=Age at first calving, SE=Standard error

**Table-2:** Analysis of variance for AFC in Frieswal cows.

Source	AFC		

	d.f.	Mean squares
Sire	56	48644.27**
Season of calving	2	38352.04
Period of calving	4	270539.76**
Error	1084	27243.04

** p<0.01

* p<0.05. AFC=Age at first calving

The least squares overall population mean of dry period was 115.29±8.83 days. This estimate was higher than the estimates reported by Singh and Gurnani; Mukherjee [13,14]. The present estimate was less than the estimates reported by Hassan and Khan; Singh [8,9] and Yadav *et al*.; Dubey and Singh [7,10]. Approximately, similar dry period i.e. 138.00±10.42, 133.00±12.30, 142.00±2.56 and 130.40±0.67 day was reported in FXJXJ crosses and in HFXS [15] and in Annual Report of Project Directorate on Cattle [12]. The season of calving had a significant influence on the dry period. Animals calved in rainy season had a shortest dry period (109.13±9.19 days) and those calved summer season had a longest dry period (118.54±9.26 days). Yadav *et al*.; Dubey and Singh [7,10] reported non-significant effect of season of calving on this trait. The period of calving was found to have a highly significant influence on dry period. However, Dubey and Singh; Hadge *et al*. [10,16] reported a significant effect of a period of calving on dry period. Results presented in Table-3 indicated that the dry period was shortest in sixth lactation and it was longest in first lactation. The statistical analysis of data showed the highly significant effect of lactation order on dry period. Singh [9] also reported a significant effect of parity order on dry period. The AFC was not found to have a non-significant influence on dry period. The overall least squares mean of calving interval was 423.05±12.24 days and this estimate was near to the values reported by Nayak and Raheja; Rathi and annual report of Project Directorate on Cattle [15,17,18]. The estimated value was more than the values reported by some workers [10,13,14,19,20]. However, higher values than the present study were reported by Yadav *et al*.; Kabir and Islam [7,21].

**Table-3:** Least square means of dry period, calving interval and service period.

Factor	Number of observation	Least squires mean		

		Dry period±SE (days)	Calving interval±SE (days)	Service period±SE (days)
Overall mean (µ)	1796	115.29±8.83	423.05±12.24	148.24±12.66
Season of calving				
Winter (November-February)	696	118.20±9.07	416.83±12.50	142.29±12.95
Summer (March-June)	501	118.54±9.26	443.11±12.70	169.59±13.17
Rainey (July-October)	599	109.13±9.19	409.21±12.62	132.83±13.08
Period of calving				
Period 1 (1993-1995)	159	107.17±11.56	423.99±15.24	142.67±15.95
Period 2 (1996-1998)	353	126.73±10.16	428.14±13.69	154.42±14.25
Period 3 (1999-2001)	784	109.76±9.04	414.97±12.46	142.79±12.91
Period 4 (2002-2004)	432	114.24±9.11	421.42±12.54	148.12±12.99
Period 5 (2005-2007)	68	118.54±12.06	426.73±15.80	153.18±16.56
Lactation order				
1	709	139.26±6.70	439.77±10.05	169.03±10.22
2	488	108.72±5.08	430.76±8.53	155.06±8.50
3	329	110.47±6.34	421.61±9.69	144.68±9.82
4	178	111.87±9.12	426.99±12.55	150.45±13.00
5	64	110.71±13.22	431.55±17.12	155.89±18.00
6	25	106.51±18.25	396.37±22.96	122.00±24.31
7 and above	3	119.50±43.15	414.31±52.80	140.55±56.33
Regressions AFC				

AFC=Age at first calving, SE=Standard error

Season of calving was found to have highly significant effect on calving interval. The highest average calving interval of 443.11±12.70 days was observed in summer season of calving, while it was lowest (409.21±12.62 days) in rainy season Hassan and Khan [8] also reported significant effect of season of calving on this trait. However, Yadav *et al*.; Dubey and Singh [7,10] reported non-significant effect of season of calving on calving interval. Period of calving was found to have non-significant effect on calving interval (Table-4). The calving interval on an average was highest (428.14±13.69 days) during second period (1996-1998) and was lowest (414.97±12.46 days) during third period (1999-2001). Singh; Dubey and Singh [9,10] also observed significant effect of period of calving on calving interval. Lactation order was found to have non-significant effect on calving interval (Table-4). Highest average calving interval of 439.77±10.05 days was found in first lactation, while lowest average calving interval of 396.37±22.96 days was found in sixth lactation. There was no definite trend in average calving interval during different lactation. Singh; Khanna and Singh; Tadesse and Dessie [9,22,23] also reported significant effect of parity order on calving interval. The AFC was found to have non-significant effect on calving interval (Table-4). The overall least squares mean of service period was 148.24±12.66 days (Table-3). Approximately same service period i.e. 143.00±11.00, 147.90±6.00, 151.07±13.23 days were reported by Singh and Gurnani; Kumar *et al*. [13,20], respectively reported the similar trend whereas the lower service period than the present finding were reported by Singh and Gurnani; Mukherjee [13,14]. Although higher estimates than the present report were also reported by Dubey and Singh; Dandapat *et al*. [10,24] in crossbred cattle. The season of calving was found to have highly significant effect (p≤0.01) on service period (Table-4). The differences among seasons may be due to the quality and availability of green fodder in different seasons. Results reflected that animals calved in summer season (March to June) had longest service period (169.59±13.17 days), whereas those calved in Rainy season (July to October) had shortest service period (132.83±13.08 days) (Table-3). Dubey and Singh [10] found no significant variations in service period due to season of calving. The period of calving had non-significant effect on service period (Table-4). The service period varied from 142.67±15.95 days during first period (i.e. 1993-1995) to 154.42±14.25 days during second period (i.e. 1996-1998) (Table-3). Significant effect of period of calving on service period was reported by Singh; Dubey and Singh [9,10]. The lactation order was found to have non-significant effect on service period. The service period on an average was highest (169.03±10.22 days) of the animals those calved first time and it was lowest (122.00±24.31 days) of the animals those calved six time (Table-3). Singh; Khanna and Singh [9,22] also observed significant effect of parity on service period in crossbred cattle. The AFC was found to have non-significant effect on service period (Table-4). Effect of sire was found non-significant on the all productive and reproductive traits under the study.

**Table-4:** Analysis of variance for dry period, calving interval and service period in Frieswal cows.

Source	d.f	Mean squares		

		Dry period	Calving interval	Service period
Sire	64	5169.83	9167.46	9991.29
Season of calving	2	14829.89*	151845.92**	174507.88**
Period of calving	4	16765.34**	9409.08	8407.96
Lactation order	6	28929.81**	7648.67	11466.76
Regressions AFC	1	2007.00	8850.21	9241.30
Error	1718	4880.66	7218.61	8242.93

** p<0.01

* p<0.05. AFC=Age at first calving

The heritability estimates of different reproduction traits in Freiswal Cattle are presented in (Table-5). In general, heritability estimates reported in the present study was a lower value. It indicates that trait is influenced by environmental variation and may be improved by minimizing the environmental variation. The heritability estimate of AFC in the present study was very low (0.15±0.065). Higher estimates of heritability than the present findings were reported by Mukherjee; Rathi; Chaudhary [14,17,25] in HF crosses. Almost similar heritability estimates were reported in an annual report [12] in HFXS. Dry period had very low heritability estimate (0.009±0.028). This estimate was comparable to those reported by Butte and Deshpande [19] in JXS. Higher estimates of heritability of dry period were reported by Chaudhary [25] in JXS, Mukherjee [14] in Friesian crosses, and low heritability, were estimated by Singh and Gurnani, Hadge *et al*.; Khan *et al*. [13,16,26] in FXT and BSXS and annual report [12] in HFXS. The heritability of calving interval in Frieswal cattle was very low (0.04±0.033), and was almost similar to those reported by Singh and Rathi [13,17,] in BSXS and Mukherjee [14] in FXS. Comparatively higher heritability were reported by Chaudhary [25] in JXS and FXS, Khan *et al*. [26] in FXS, Dubey and Singh [10] in SXCB and Singh [11]. The low heritability estimate of service period (0.03±0.032), revealed that the service period of Frieswal may further be improved by improving feeding and management including proper heat detection, timely insemination, and pregnancy diagnosis, and estimate was similar to reported by Mukherjee [14] in FXS. However, higher heritability estimates were reported by Dubey and Singh [10] in SXCB.

**Table-5:** Heritability estimate of different reproduction traits in Frieswal cows.

Trait	Heritability (h^2^±SE)
AFC	0.15±0.065
Dry period	0.009±0.028
Calving interval	0.04±0.033
Service period	0.03±0.032

AFC=Age at first calving, SE=Standard error

The present study pertains to the estimation of genotypic and phenotypic correlations among different reproduction traits with a view to investigate whether any association exists between the reproductive traits in Freiswal Cattle maintained at Military Dairy farm Ambala. The correlation between two traits was estimated by the method of analysis of variance and covariance by paternal half-sib analysis. The genetic correlation between two characters arises due to the pleiotropic effect of gene and some linkage among genes. However, in a large population under random mating, the effect of linkage in quantitative genes is expected to be negligible. Phenotypic correlations between two traits are a function of genetic and environmental correlations between them with the assumption that there is no covariance between genotype and environment. The genetic and phenotypic correlation coefficients among various reproductive traits are presented in (Table-6). Service period was found to have a high positive genetic correlation with dry period and calving interval. The corresponding estimates were 0.16±1.24 and 0.91±0.10. Dubey and Singh [10] reported medium (0.57±0.14), Saha [27] reported low (0.18±0.28) genetic correlation between service period and dry period. Singh and Gurnani [13] also found very high (0.91±0.13) genetic correlation between service period and calving interval. The phenotypic correlations with dry period (0.61) and calving interval (0.93) were high and was similar to as reported by Singh and Gurnani; Saha [13,27].

**Table-6:** Genetic (r_g_±se) (above diagonal) and phenotypic (r_p_) (below diagonal) correlations among service period, dry period and calving interval in Frieswal cows.

Parameter	Service period	Dry period	Calving interval
Service period	-	0.161±1.246	0.910±0.102
Dry period	0.612	-	0.137±1.162
Calving interval	0.934	0.672	-

## Conclusion

The average AFC was found to be a significant difference in different periods. The highest dry period was noted in summer season while the rainy season had the lowest. The calving interval proved to be dependent in its expression on year, the season of calving and parity of cow. The season of calving was found to have a highly significant effect on service period, and there was no effected by period of calving and parity of cow. In general, the heritabilities for all reproductive traits with the exception of AFC were low. From the results of this study, it may be concluded that proper feeding and management is necessary for the improvement of reproductive traits of Frieswal.

## Authors’ Contributions

YPS planned the study. JK recorded the information and analyzed the data. SK, RS, RK, and PK provided help in analyzing the data. JK drafted and revised the manuscript under the guidance of SK and YPS. All authors read and approved the final manuscript.
